# Uses of Antibiotics in Ornamental Fish in Hong Kong and the Antibiotic Resistance in the Associated Zoonotic Pathogens

**DOI:** 10.3390/jox12040026

**Published:** 2022-12-04

**Authors:** Chun Au-Yeung, Kit-Ling Lam, Ka-Wai Chan, Wing-Yin Mo

**Affiliations:** Department of Applied Science, School of Science and Technology, Hong Kong Metropolitan University, Ho Man Tin, Hong Kong

**Keywords:** antibiotic residues, tetracyclines, fluoroquinolones, *Aeromonas* spp., *Pseudomonas* spp., minimum inhibitory concentration

## Abstract

The use of antibiotics in ornamental fish is not regulated, as they are not intended for human consumption. Although antibiotic resistant bacteria have been detected in ornamental fish worldwide, there have been no studies to look at the situation in Hong Kong. Therefore, the present study was conducted to investigate the use of antibiotics in ornamental fish. Ornamental fish were purchased from five local pet fish shops and the antibiotics in carriage water were quantified using liquid chromatography tandem mass spectrometry. Moreover, *Aeromonas* and *Pseudomonas* spp. present in carriage water were isolated and their minimum inhibitory concentrations against selected antibiotics were determined. Results indicated that among the twenty antibiotics screened, doxycycline (0.0155–0.0836 µg L^−1^), oxytetracycline (0.0102–29.0 µg L^−1^), tetracycline (0.0350–0.244 µg L^−1^), enrofloxacin (0.00107–0.247 µg L^−1^), and oxalinic acid (n.d.−0.514 µg L^−1^) were detected in all sampled shops. Additionally, MIC results revealed that some of the *Aeromonas* and *Pseudomonas* spp. isolates were highly resistant to all antibiotics selected. Our findings confirmed that multiple antibiotics are being used in ornamental fish and the associated bacteria are resistant to selected antibiotics, suggesting that this could be a significant transmission route of antibiotic resistant bacteria to household indoor environments.

## 1. Introduction

The live ornamental fish trade is a rapidly growing sector of the aquaculture industry worldwide. The market size for the industry was estimated at USD 5.4 billion in 2021, and it has been predicted to increase by 8.5% from 2022 to 2030 [1]. To maximize productivity, intensive cultivation systems have been adopted in ornamental fish farms, creating stressful conditions such as high stocking density and suboptimal hygiene, which would weaken host defenses and increase incidences of microbial diseases such as columnaris and furunculosis [2,3]. Antibiotics have become the primary means of reducing loss caused by bacterial infection [4]. Antibiotics have been commonly administered in the form of medicated feed or baths as growth promotors, disease treatment, or prophylactic measures to prevent opportunistic pathogens during cultivation or transportation [5,6]. A wide range of antibiotics and chemotherapeutics have been indiscriminately applied in Chinese aquaculture, such as fluoroquinolones, macrolides, sulfonamides, and tetracyclines [7,8]. To our knowledge, rather limited studies have been conducted to investigate antibiotic use in the ornamental fish industry. A recent study carried out in Sri Lanka reported the heavy use of antibiotics in a local ornamental fish farm [9]. Antibiotic usage in food fish has been extensively studied and reported. Oxytetracycline, sulfonamides, and quinolones are some of the most common antibiotic and chemotherapeutic agents used worldwide in food fish [10]. Similar antibiotics are utilized in ornamental fish farming as well as food fish because of their broad-spectrum bacteriostatic properties against gram-negative bacteria [7,8,11,12]. Two studies on antibiotic residues in the muscle tissue of cultured fish in China revealed a high prevalence and residual concentrations of tetracyclines, with similar concentrations detected [8,12]. As the regulations on food fish do not apply to ornamental fish, it is reasonable to predict that the use of antibiotics in the ornamental sector is even more prevalent.

It has been suggested that ingested antimicrobial agents are poorly absorbed by fish and are eventually excreted with their metabolites in feces into the environment with their antimicrobial activity intact [13,14,15]. The discharge of these antimicrobials into the aquatic environment exerts selective pressures, creating reservoirs of antibiotic-resistant bacteria (ARB) and transferrable resistance genes in both fish pathogens and normal flora in the water body. As a result, the indigenous microbial community in the environment will be disrupted and selected, resulting in a decrease in microbial diversity [16,17,18]. Apart from microbial selection, antibiotic resistance genes have been exchanged between bacteria of terrestrial and aquatic origin [19]. The acquisition of antibiotic resistance through horizontal gene transfer events increases the incidence of drug-resistance, thereby posing a significant threat to human health [20]. Numerous investigations from mainland China and other countries have shed light on the occurrence and persistence of antibiotic resistance in aquaculture systems, including those for pet and food fish [21,22,23,24,25,26]. On the contrary, in Hong Kong, there are no environmental surveillance programs or regulations regarding antimicrobial use in the ornamental fish industry. To our best knowledge, the application of antibiotics in ornamental species is largely unknown, and there is no published study to investigate the types and concentrations of antibiotics. Therefore, the amount of antimicrobial use in ornamental fish is indiscriminate, often without prescription. Abuse of antimicrobials exposes the farming environment to various antimicrobials and poses a huge challenge to the fish and microbial communities [27].

Zoonotic pathogens isolated in ornamental fish that exhibit resistance to various antibiotics have been reported [5,22,27,28]. Given that zoonotic diseases associated with ornamental fish have been reported in humans, there is a possibility of infection from ornamental fish to humans, as well as a risk of ARB transmission to the general public [5]. Certain classes of antibiotics, which include fluoroquinolones and macrolides, are regarded as crucial for disease control in humans [29]. Increased exposure to antibiotics used only in human medicine potentiates the emergence of drug-resistant bacteria, which in turn compromises human health by reducing the number of available and effective treatments [30]. Therefore, the situation of antibiotic use and resistant bacteria in the ornamental fish industry should be investigated. It is also important for the authorities, the public, and the industry to recognize the extent to which antibiotic use associated with ornamental fish has expedited the spread of antibiotic resistance.

This work aims to identify and quantify the antibiotics used in ornamental fish available in Hong Kong and to study the resistance of zoonotic pathogens associated with ornamental fish to selected antibiotics. Given the historical use of antibiotics such as oxytetracycline and oxalinic acid in aquaculture, it is likely that high levels of antibiotic residues contribute to the development of antibiotic tolerance and the dissemination of antibiotic resistance in the ornamental fish industry [22]. *Aeromonas* and *Pseudomonas* spp. are zoonotic bacteria and ubiquitous in aquatic environments, and exposure to these bacteria could be a potential transmission route of drug-resistant bacteria between humans and ornamental fish [31,32]. Therefore, their susceptibilities to selected antibiotics were also studied in the form of minimum inhibitory concentrations (MIC).

## 2. Materials and Methods

### 2.1. Chemicals and Reagents

Methanol (MeOH) and acetonitrile (ACN) were of HPLC grade and purchased from Duksan Company (Ansan, Republic of Korea). Analytical-grade formic acid, sodium hydroxide and ammonium acetate were provided by Fisher Scientific (Waltham, WA, USA) and Fluka (Buchs, Switzerland), respectively. Milli-Q water from Merk Millipore was used throughout the study when deionized water was needed. Twenty native antibiotic standards with purity > 98% used in solid-phase extraction-liquid chromatography-tandem mass spectrometry (SPE-LC-MS/MS) analysis belonged to five groups of antibiotic, including four tetracyclines [tetracycline (TC), chlortetracycline (CTC), oxytetracycline (OCT), tetracycline (TC)], six fluoroquinolones (ciprofloxacin (CFX), enrofloxacin (EFX), ofloxacin (OFX), oxalinic acid (OA), sparfloxacin (SAR), and sarafloxacin (SFX)], three macrolides [clarithromycin (CTM), roxithromycin (RTM), and tylosin (TYL)], and six sulfonamides [sulfadiazine (SDZ), sulfamethazine (STZ), sulfamonomethoxine (SMM), sulfathiazole (SAZ), sulfamethoxazole (SMX), and sulfamerazine (SMZ)], and a diaminopyrimidines [trimethoprim (TMP)], were purchased from Sigma-Aldrich (St. Louis, MO, USA) and J&K Chemical Ltd. (Beijing, China). The internal standards, including sulfamethoxazole-13C6, ciprofloxacin-d8, roxithromycin-d7, and caffeine-13C3, were purchased from HPC Standard GmbH (Cunnersdorf, Germany) and Cambridge Isotope Laboratory (Tewksbury, MA, USA). The stock solution of individual compounds and internal standards (1 g L^−1^) was prepared by dissolving 10 mg in 10 mL of methanol for all antibiotics except fluoroquinolones, which was dissolved in methanol with sodium hydroxide. All stock solutions were stored at −80 °C. A 0.5 mg L^−1^ mixture of working standard containing all native compounds was freshly prepared by diluting the stock solution with methanol.

All antibiotics used in microbiological analyses were of high purity (>95%). Tetracycline hydrochloride, doxycycline hydrochloride, oxytetracycline hydrochloride, enrofloxacin, and oxalinic acid were purchased from Santa Cruz Biotechnology (Heidelberg, Germany) and MedChemExpress (Monmouth Junction, NJ, USA). Bacteria culturing media used in this study include nutrient broth (BD Difco, Sparks, MD, USA), nutrient agar (Oxoid, Hampshire, UK) and cation-adjusted Mueller Hinton broth (CAMHB) (Sigma-Aldrich, St. Louis, MO, USA). In addition, glutamate starch phenol red agar (GSP agar) was prepared according to the recipe provided by Sigma-Aldrich [33].

### 2.2. Research Premises and Sampling

Freshwater ornamental fish were purchased at a monthly interval evenly split between August and September 2022 from five different pet fish shops (S1–S5) in Goldfish Market, Kowloon. In each shop, three packs of ornamental fish (together with the carriage water) were purchased each time, and thirty samples were purchased for the study. Out of the thirty samples collected, twelve each were of zebrafish (*Danio rerio*) and southern platyfish (*Xiphophorus maculatus*), and six were of koi carp (*Cyprinus rubrofuscus*). Samples were immediately transported to the laboratory. Fish carriage water was divided into two portions for antibiotic concentration analysis (300 mL) and bacterial analysis (1 mL), according to Section 2.3 and Section 2.4, respectively.

### 2.3. Antibiotic Analysis

#### 2.3.1. Sample Extraction

Antibiotics in 30 carriage water subsamples were extracted and quantified using solid-phase extraction (SPE) coupled with liquid chromatography-tandem mass spectrometry (LC-MS/MS) according to Chen and Zhou with some modifications [34]. Briefly, 300 mL of carriage water samples were adjusted to pH 3.5 with 50% formic acid and spiked with 50 µL of 1000 µg L^−1^ internal standards. Acidified samples were cleaned up and concentrated by SPE using an Oasis HLB cartridge (6 cc, 200 mg sorbent, Waters^®^, Milford, MA, USA). SPE cartridge was preconditioned with 6 mL methanol, followed by 12 mL Milli-Q water and 6 mL Milli-Q water adjusted to pH 3.5 ± 0.05 with 50% formic acid, before the water samples were percolated at a flow rate of 5 mL min^−1^. After extraction, antibiotics were eluted from the SPE cartridge with 8 mL of methanol, followed by concentrating 0.2 mL under a gentle nitrogen stream. Finally, the extracts were reconstituted to 1 mL using water and methanol (8:2, *v/v*).

#### 2.3.2. Instrumental Analysis

An Agilent 1290 infinity LC system coupled with an Agilent 6460 triple quadrupole mass spectrometer (Agilent, Palo Alto, CA, USA) equipped with positive electrospray ionization (ESI) mode was used to determine all target antibiotics. The mass spectrometric condition was as follows: capillary voltage: 4000 V, nebulizer pressure: 45 PSI, drying gases temperature: 350 °C, and source gas flow: 10 L min^−1^. Analyte separation was achieved by injecting 10 µL sample into an Agilent InfinityLab Poroshell 120 EC-C18 (3.0 150 mm 2.7 µm) with guard filter. The mobile phases used in the chromatography included phase A: 0.1% formic acid with 5 mM ammonia acetate in Milli-Q water and phase B: ACN: MeOH 80:20 (*v/v*). The mobile phase gradient was performed at a flow rate of 0.4 mL min^−1^ from 95% A for 2 min, 60% A for 5 min, 55% A for 2 min, 40% A for 1 min, 95% A for 2 min, and finally, hold 95% A for 0.5 min. Each run lasted for 12.5 min and was followed by a 3-min post-run. The protonated ion ([M+H]^+^) was selected for the mass spectrometer analysis. LC-MSMS method was established after extensive optimization. The detailed optimization conditions, including retention time, precursor ion, product ions, and fragmentation are shown in Table A1.

#### 2.3.3. Method Validation and Quality Control

In order to evaluate the accuracy and sensitivity of the analytical method, recovery, limit of detection (LOD), and limit of quantification (LOQ) were studied. Recoveries were calculated by spiking both native antibiotics and internal standards into Milli-Q water in triplicate at concentrations of 50 and 100 ng L^−1^. The recoveries of the target compounds were between 65% and 118% and 58.9% and 99.3%, respectively. The limits of quantification were calculated based on the standard deviation of response and the slope of each compound, ranged from 0.03 to 0.95 ng L^−1^, while the limits of detection ranged from 0.01 to 0.31 ng L^−1^. (Details are shown in Table A2). The concentration of target antibiotics in water samples was quantified using the internal standard method in order to compensate for the matrix effects between the samples. Isotope labeled internal standards were spiked in samples and the calibration curve was set at the same concentration. A calibration curve was constructed from seven points spiked with internal standards (1, 5, 10, 20, 50, 75, 100 µg L^−1^). The determination coefficients of the calibration curve varied from 0.98 to 0.99. Furthermore, the procedural blank was run to check for contamination, and a quality control sample was run every ten samples, followed by an injection of the solvent blank.

### 2.4. Bacterial Analysis

#### 2.4.1. Bacterial Isolation

One hundred microliters of water samples (10^0^, 10^−1^ and 10^−2^) were spread on GSP agar in duplicate to isolate *Aeromonas* and *Pseudomonas* spp. The agar plates were incubated aerobically at 30 °C for 48–72 h. Subsequently, plates were inspected, and colony numbers were counted based on morphological characteristics, according to pigmentation, colony form, and surface appearance. Yellow colonies surrounded by a yellow zone were presumed to be *Aeromonas* spp., while the blue-violet colonies surrounded by a red-violet zone were presumed to be *Pseudomonas* spp. Four colonies of each target bacterium isolated from each shop in two months were randomly selected for antibiotic susceptibility test. Reference strains *Aeromonas hydrophila* ATCC 7966, *Escherichia coli* ATCC 25922 and *Pseudomonas aeruginosa* ATCC 10145 were used as quality control.

#### 2.4.2. Antibiotic Susceptibility Test

Antimicrobial susceptibility testing was performed by a standard two-fold serial broth microdilution method using CAMHB according to the guidelines of the Clinical and Laboratory Standards Institute (CLSI) [35,36]. Prior to sampling in August and September 2022, preliminary work was conducted to determine the most commonly used antibiotics. The preliminary results (data not shown) suggested that DC, OTC, TC, EFX, and OA were the major antibiotics detected in the carriage water samples. These antibiotics were also selected for the subsequent antibiotic susceptibility study.

Four single colonies of presumptive *Aeromonas* spp. and *Pseudomonas* spp. isolated from GSP agar were selected randomly. The minimum inhibitory concentrations (MICs) of these colonies were determined against five antibiotics: DC, TC, OTC, EFX and OA. Each colony was suspended in 3 mL phosphate-buffered saline and adjusted to 0.5 McFarland standard with a nephelometer (V3011, Thermo Scientific, MA, USA). The bacterial suspension containing about 1 × 10^8^ CFU mL^−1^ was diluted to 1 × 10^6^ CFU mL^−1^ with CAMHB. Fifty microliters of the bacterial suspension were added to each well of a 96-well plate (Jet Biofil, Guangzhou, China). Each well of the 96-well plate contained antibiotic solution at final concentrations as follows: doxycycline, 8–128 mg L^−1^; tetracycline 2–256 mg L^−1^; oxytetracycline 2–256 mg L^−1^; enrofloxacin 0.5–64 mg L^−1^; and oxalinic acid 0.5–32 mg L^−1^. The plate was incubated at 37 °C for 18 h and the absorbance at 600 nm was measured.

The MIC of each isolate for each compound was determined as the lowest concentration of an antimicrobial that inhibited the growth of a given culture. Each isolate was examined in triplicate, and each batch of media was checked with *E. coli* ATCC 25922. The susceptibility of isolates was interpreted following the guidelines of CLSI and the breakpoint of each antibiotic as follows: doxycycline, 8 mg L^−1^; oxytetracycline, 64 mg L^−1^; tetracycline, 16 mg L^−1^; enrofloxacin, 0.5 mg L^−1^; and oxalinic acid, 0.5 mg L^−1^ [36,37,38,39]. The MIC values obtained were used to determine the mean MIC values, ranges of MIC, and resistant prevalence. Resistant prevalence was only conducted for *Aeromonas* spp., owing to the availability of epidemiological cut-off values and antibiotic susceptibility studies related to aquaculture [36,40,41,42].

### 2.5. Data Analysis

The mean MIC values of *Aeromonas* and *Pseudomonas* spp. isolated from the five sampled shops in two months were determined to represent the overall antibiotic resistance. If the MIC of the isolate was lower or higher than the corresponding test range of each antibiotic, a two-fold decrease or increase of the minimum or maximum test point was used in the calculation, respectively. The relationship between antibiotic concentrations in carriage water and the mean MIC value of *Aeromonas* and *Pseudomonas* spp. isolates from the same sample was examined by Pearson correlation. All analyses were performed in Prism 9.4.1 for Mac (GraphPad Software, San Diego, CA, USA).

## 3. Results

### 3.1. Concentrations of Antibiotics in Carriage Water Samples

Concentrations of antibiotics detected in the carriage water samples are summarized in Table 1. Of the twenty antibiotics tested, all MLs, SAs, one TCs (CTC), and five FQs (CFX, OFX, NFX, SAR and SFX) were not detected in all carriage water samples. Five antibiotic compounds, including three TCs (DR, OTC and TC) and two FQs (EFX and OA), were detected in carriage water samples collected in August. Meanwhile, for the samples collected in September, three TCs (DC, OTC, and TC) and one FQs (EFX) were detected. In general, OTC was observed to be the most dominant compound from all carriage water samples collected in two months from five shops, with the concentration ranging from 0.102 to 29.0 µg L^−1^.

### 3.2. Antibiotic Resistance Prevalence and MIC of Aeromonas and Pseudomonas spp.

Table 2 and Table 3 show the prevalence of antibiotic resistant isolates and MIC ranges and mean MIC concentrations of each sample collected from two sampling months in five shops for the five antibiotics for *Aeromonas* and *Pseudomonas* spp., respectively. All *Aeromonas* isolates displayed high resistance to OTC, TC and OA, with a resistance prevalence rate above 90%. This result indicates that most of the *Aeromonas* spp. isolates were resistant to OTC, TC, and OA. The resistance rates for DC and EFX ranged from 55% to 70%, respectively, indicating a moderate susceptibility to these two compounds. As there are no reported MIC cut-off values for *Pseudomonas* spp., the prevalence of susceptibility could not be assessed. Resistance of the *Pseudomonas* isolates to OTC and TC was high, with the MIC levels not less than 32 mg L^−1^, whereas resistance to DC, EFX, and OA was low to moderate. Overall, the resistance levels of the two target bacteria among sampling shops differed considerably, with the exception of the consistently high MIC levels of OTC and TC, and the MICs of isolates from the two months displaying similar levels. It should be noted that the highest MIC for OTC and TC against isolated *Aeromonas* spp. and *Pseudomonas* spp. were ≥256 mg L^−1^.

### 3.3. Correlations of Concentrations of Antibiotics and the Corresponding MIC Levels of Aeromonas and Pseduomonas spp.

**Figure 1:**
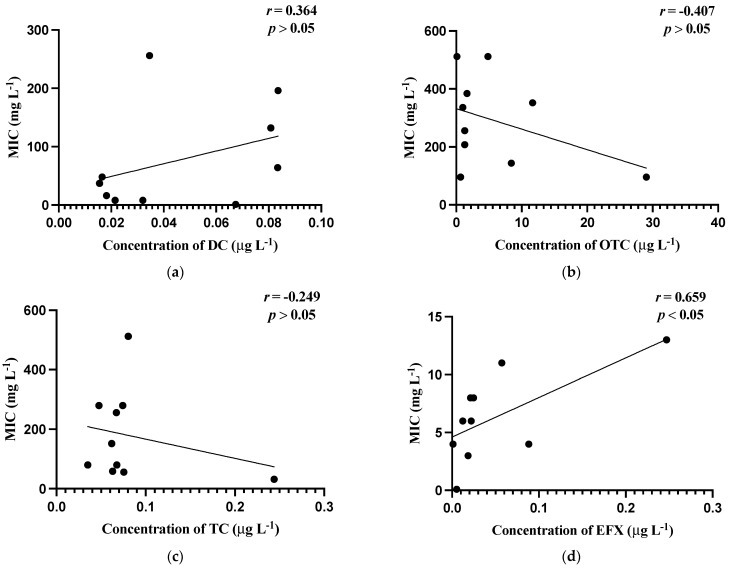

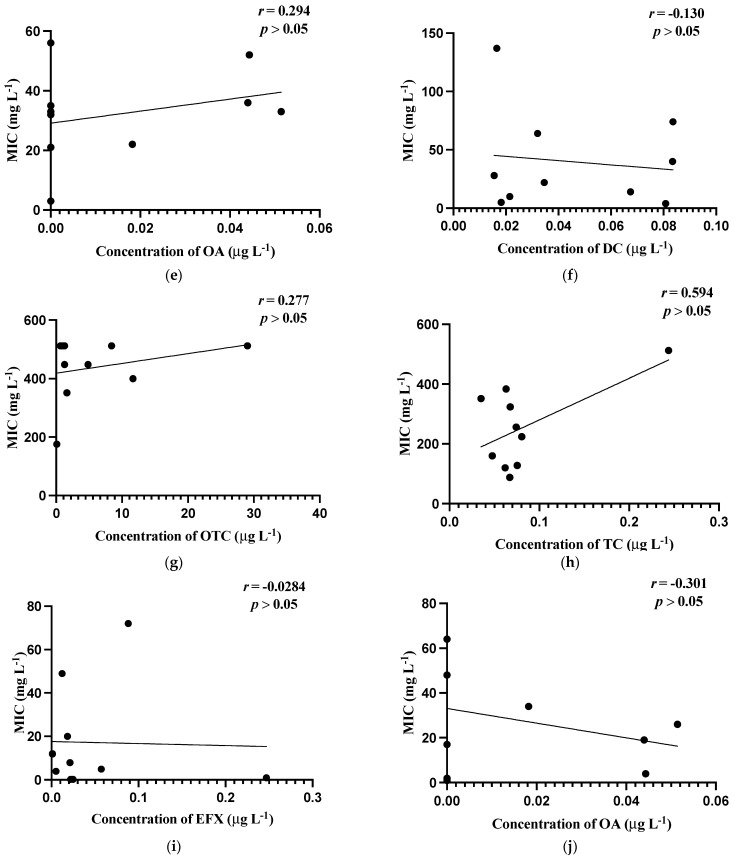
Correlations of minimum inhibitory concentrations (MICs, mg L^−1^) against the residual concentrations of antibiotics (µg L^−1^). (**a**) MIC (mg L^−1^) of *Aeromonas* spp. against concentration (µg L^−1^) of doxycycline; (**b**) MIC (mg L^−1^) of *Aeromonas* spp. against concentration (µg L^−1^) of oxytetracycline; (**c**) MIC (mg L^−^^1^) of *Aeromonas* spp. against concentration (µg L^−1^) of tetracycline; (**d**) MIC (mg L^−1^) of *Aeromonas* spp. against concentrations (µg L^−1^) of enrofloxacin; (**e**) MIC (mg L^−1^) of *Aeromonas* spp. against concentrations (µg L^−^^1^) of oxalinic acid; (**f**) MIC (mg L^−1^) of *Pseudomonas* spp. against concentrations (µg L^−1^) of doxycycline; (**g**) MIC (mg L^−1^) of *Pseudomonas* spp. against concentrations (µg L^−1^) of oxytetracycline; (**h**) MIC (mg L^−1^) of *Pseudomonas* s spp. against concentrations (µg L^−1^) of tetracycline; (**i**) MIC (mg L^−1^) of *Pseudomonas* spp. against concentrations (µg L^−^^1^) of enrofloxacin; (**j**) MIC (mg L^−^^1^) of *Pseudomonas* spp. against concentrations (µg L^−^^1^) of oxalinic acid.

Figure 1 shows the relationship between mean MIC values of bacterial isolates and the mean concentrations of the antibiotics detected in the carriage water samples. The values of *Aeromonas* spp. Figure 1a–e utilized were obtained by excluding values below the breakpoints, whereas those of *Pseudomonas* spp. Figure 1f–j were calculated using the means after excluding values below the test range.

In general, weak relationships were observed between MIC levels and the concentrations of antibiotics in carriage water samples, which were reflected by the low correlation coefficient (*r*) values obtained. Noticeably, only the correlation between the MIC values of *Aeromonas* isolates and the concentration of EFX in the carriage water samples was the strongest and significant (*p* ≤ 0.05).

## 4. Discussion

To our knowledge, this is the first study that has investigated antibiotic consumption in ornamental fish and antibiotic resistance in *Aeromonas* and *Pseudomonas* spp. in Hong Kong. The Goldfish Market is a popular tourist spot, and it is also a place where ornamental fish shops are concentrated, with 40 to 50 shops selling a large variety of ornamental fish. Most of the ornamental fish are imported from other regions and immediately after arrival, the fish will be introduced to fish tanks. Fish sold in the Goldfish Market are either pre-packaged in plastic bags and then hung at the entrance of stores or displayed in fish tanks for selection by customers. These display methods are stressful to fish, creating favorable conditions for disease prevalence. Therefore, it is unsurprising that antibiotics are added to rearing or carriage water to ensure the fish’s survival before sale. Among the 20 antibiotics analyzed, DC, OTC, TC, EFX, and OA were most commonly detected during the study period. Except for OA, four antibiotics (DC, OTC, TC, and EFX) were detected in all carriage water samples, suggesting that antibiotics were being supplemented regularly in fish tanks in all shops.

In Hong Kong, the regulations on antibiotic use only apply to food animals. For example, no more than 100 µg kg^−1^ of oxytetracycline is permitted [43]. Restrictions regarding the ornamental fish industry have yet to be established by the government, as well as the antibiotic use and susceptibility study for the industry. Currently, no research regarding antibiotic use and the corresponding susceptibility has been established for the ornamental fish industry, nor has the government implemented any surveillance programs or regulations in order to understand and monitor the local situation.

The concentrations of antibiotic residues in carriage water samples from the ornamental fish market varied by shops and months. The quantitative variation in the occurrence of antibiotics in the carriage water samples can be attributed to different practices and needs in the rearing of ornamental fish with regard to the use of antibiotics. For instance, oxytetracycline and enrofloxacin had the highest concentrations in the class of tetracyclines and fluoroquinolones, ranging from 0.102 to 29.0 µg L^−1^ and 0.00107 to 0.247 µg L^−1^, respectively. In addition, the widespread use of oxytetracycline and enrofloxacin in the industry can also be observed. This observation, in terms of the use of antibiotics in rearing ornamental fish, is similar to the findings of a previous investigation conducted in Sri Lanka [9].

Due to the large proportion of antibiotic compounds that can enter and persist in aquatic environments, they will have the potential to select for resistant bacteria in the environment over time [4]. The transmission of antibiotic resistance genes from microorganisms in the aquatic environment to terrestrial microbial species will eventually reach human consumers. Food fish have been recognized as one of the major reservoirs of ARB and resistance genes [44]. Knowing that a large variety of antibiotic residues have been recorded in aquatic products, the microbiome of aquatic products may have undergone selection and developed resistance to different antibiotics [7]. Among the twenty antibiotics targeted in this study, OA and EFX are exclusively used for veterinary purposes, while DC, OTC and TC are used in both humans and animals [7,29]. The high frequency of detection of bacteria that are resistant to OTC and TC in the present study suggested that the ornamental fish industry may also be a hotspot for the emergence of antibiotic resistance. Previous studies also reported a high prevalence of resistance to common antibiotics, including OTC and TC used in ornamental fish available in different locations, demonstrating that the ornamental fish industry is a potential reservoir of multi-drug resistant bacteria [45,46,47,48]. In addition to ARB, the prevalence of resistance genes has been investigated worldwide [10]. The wastewater discharged from an ornamental fish market in China was found to contain high levels and a wide range of resistance genes, showing the potential of ornamental fish to be a reservoir of antibiotic residues [21].

Nevertheless, the present study confirmed that antibiotics are being used by the ornamental fish sellers in Hong Kong, and rather high resistance was observed in the bacteria present in the carriage water samples. The bacteria associated with fish could also be the hotspots of ARB. Moreover, Figure 1 shows weak correlations established between the majority of antibiotic residues and corresponding MIC levels in the present study. The presence of high levels of ARB with low concentrations of antibiotics was detected. One reason is probably that antibiotic resistance was developed in ornamental fish farms prior to their distribution to the retail market.

The detection of zoonotic pathogens with high resistance to multiple antibiotics in fish carriage water is rather alarming. The prophylactic use of antibiotics in ornamental fish should be discontinued to reduce the development of ARB. Reducing the stocking density of fish, for example, is one of the simple but effective ways to reduce the stress experienced by fish in the display tanks, which in turn reduces the need for antibiotics. Although ornamental fish are not intended for human consumption, the bacteria could persist in household fish tanks for an extended period and be transmitted to humans. Furthermore, aeration by an air pump in a household aquarium can be a means to transmit pathogens from one tank to another via aerosols [49,50]. Aerosolized ARB will contaminate the surrounding environment and potentially be inhaled by humans [51]. As fishkeeping is one of the most popular hobbies in Hong Kong, introducing fish that carry ARB could pose potential health risks to citizens. The potential role of fish tanks in the dissemination of ARB will be investigated in the future.

## 5. Conclusions

In this work, the quantity of antibiotics and ARB prevalence in the carriage water of ornamental fish were studied. OTC had the highest concentration of antibiotics detected during the two-month sampling period in all sampled shops. Moreover, high levels of antibiotic resistance were found in the susceptibility tests of Aeromonas and Pseudomonas spp. against OTC and TC. There were low or no positive correlations between the MIC levels of the two target bacteria and concentrations of antibiotic residues, suggesting that additional variables may contribute to the development of antibiotic resistance in the local ornamental fish industry. It is reasonable to assume that high levels of antibiotic resistance were developed before arriving the sampled shops.

## Figures and Tables

**Table 1:** The concentrations of antibiotics detected in carriage water samples collected from Goldfish Market in two months (mean ± SD, *n* = 15).

Carriage Water Samples		Concentration (µg L^−1^) of Antibiotic				
		Tetracyclines			Fluoroquinolones	
		DC	OTC	TC	EFX	OA
S1	Aug	0.0834 ± 0.0153	0.999 ± 0.262	0.0743 ± 0.00153	0.247 ± 0.0306	0.0443 ± 0.000351
	Sep	0.0165 ± 0.00153	11.6 ± 1.73	0.0676 ± 0.00839	0.0883 ± 0.0121	N.D.
S2	Aug	0.0836 ± 0.00231	4.82 ± 1.86	0.0805 ± 0.00379	0.0572 ± 0.00551	0.440 ± 0.000208
	Sep	0.0346 ± 0.00643	1.64 ± 1.04	0.0476 ± 0.0110	0.00107 ± 0.00134	N.D.
S3	Aug	0.0808 ± 0.00116	0.102 ± 0.00577	0.0670 ± 0.00173	0.0122 ± 0.00115	0.0514 ± 0.000361
	Sep	0.0182 ± 0.00153	8.42 ± 2.11	0.0754 ± 0.0187	0.0221 ± 0.00173	N.D.
S4	Aug	0.0320 ± 0.00770	0.649 ± 0.161	0.0630 ± 0.0330	0.0212 ± 0.00954	0.0182 ± 0.000557
	Sep	0.0215 ± 0.00730	1.29 ± 0.466	0.0350 ± 0.00954	0.0247 ± 0.0101	N.D.
S5	Aug	0.0155 ± 0.000577	29.0 ± 9.10	0.244 ± 0.0844	0.0184 ± 0.000153	N.D.
	Sep	0.0674 ± 0.0104	1.28 ± 0.790	0.0619 ± 0.0267	0.00524 ± 0.00127	N.D.

Note: Aug = August 2022; Sep = September 2022; N.D., not detected; DC = doxycycline; OTC = oxytetracycline; TC = tetracycline; EFX = enrofloxacin; OA = oxalinic acid; S1–S5 = the five shops sampled.

**Table 2:** Antibiotic resistance prevalence and levels of *Aeromonas* spp.

Antibiotics		N (%)	MIC Range (mg L^−1^)	MIC Breakpoint (mg L^−1^)	Sampled Shops									
					S1		S2		S3		S4		S5	
					N	Mean of MIC (mg L^−1^)	N	Mean of MIC (mg L^−1^)	N	Mean of MIC (mg L^−1^)	N	Mean of MIC (mg L^−1^)	N	Mean of MIC (mg L^−1^)
DC	Aug	22 (55)	≤8–≥128	8	1	64	4	196	4	132	2	8	3	37
	Sep				2	48	2	256	1	16	3	8	0	0
OTC	Aug	37 (94.9)	32–≥256	64	4	336	4	512	3	512	2	96	4	96
	Sep				4	352	4	384	4	144	4	256	4	208
TC	Aug	38 (97.4)	16–≥256	16	4	280	4	512	3	256	3	59	4	32
	Sep				4	80	4	280	4	56	4	80	4	152
EFX	Aug	28 (70)	≤0.5–32	0.5	4	13	4	11	3	6	1	8	3	3
	Sep				2	4	3	4	4	6	4	8	0	0
OA	Aug	38 (95)	≤0.5–≥32	0.5	4	52	4	36	4	33	3	22	3	35
	Sep				4	33	4	21	4	32	4	56	4	3

Note: Aug = August; Sep = September; N (%) = Resistance prevalence number of *Aeromonas* spp. (Resistance prevalence rate); MIC = minimum inhibitory concentration; DC = doxycycline, OTC = oxytetracycline; TC = tetracycline; EFX = enrofloxacin; OA = oxalinic acid; S1–S5 = the five shops sampled.

**Table 3:** Antibiotic resistance prevalence and levels of *Pseudomonas* spp.

Antibiotics		N	MIC Range (mg L^−1^)	Sampled Shops				
				S1	S2	S3	S4	S5
				Mean of MIC (mg L^−1^)				
DC	Aug	40	≤8–≥128	40	74	4	64	28
	Sep			137	22	5	10	14
OTC	Aug	40	64–≥256	512	448	176	512	512
	Sep			400	352	512	512	448
TC	Aug	40	32–≥256	256	224	88	384	512
	Sep			324	160	128	352	120
EFX	Aug	40	≤0.5–≥64	1	5	49	8	20
	Sep			72	12	0.25	0.25	4
OA	Aug	40	≤0.5–≥32	4	19	26	34	64
	Sep			64	48	1	2	17

Note: Aug = August 2022; Sep = September 2022; N = Total *Pseudomonas* isolates; MIC = minimum inhibitory concentration; DC = doxycycline, OTC = oxytetracycline; TC = tetracycline; EFX = enrofloxacin; OA = oxalinic acid; S1–S5 = the five shops sampled.

## Data Availability

Not applicable.

## Appendix A

**Table A1:** Optimized LC-MS/MS parameters including retention time, precursor, product ions, and fragmentation.

Target Antibiotics	Abbreviation	Retention Time (min)	Precursor Ion	Product Ion 1	Product Ion 2	Fragmentation (v)
Ciprofloxacin	CFX	3.85	332.1	231.1	314.1	135
Enrofloxacin	EFX	4.013	360.2	342.2	316.2	135
Ofloxacin	OFX	3.815	362.2	318.2	261.1	145
Oxalinic acid	OA	6.213	262.1	216	244.1	85
Sparfloxacin	SAR	4.328	393.2	349.2	251.1	150
Sarafloxacin	SFX	4.314	386.1	368.2	342.2	150
Norfloxacin	NFX	3.80	320.1	302.2	231.1	105
Chlortetracycline	CTC	4.926	479.1	462.2	444.1	150
Doxycycline	DC	5.152	445.2	428.1	98	125
Oxytetracycline	OTC	3.919	461.2	426.1	443.2	140
Tetracycline	TC	4.089	445.1	410.1	154	120
Tylosin tartrate	TYL	8.625	916.5	174.1	101.1	185
Roxithromycin	RTM	11.545	839.5	158.1	116.1	190
Clarithromycin	CTM	11.382	748.5	158.1	116	200
sulfamethazine	STZ	4.496	279.1	186	124.1	115
sulfamonomethoxine	SMM	4.915	281.1	156	108	105
sulfathiazole	SAZ	3.898	256	156	92.1	110
sulfamethoxazole	SMX	5.564	254.1	156	92	110
Sulfamerazine	SMZ	4.193	265.1	108	92.1	110
Sulfadiazine	SDZ	3.865	251.1	108.1	92.1	120
Trimethoprim	TMP	5.784	291.2	230.1	123	155
Sulfamethoxazole-13C6	SMZ-13C6	5.557	261.1	162	98	87
Ciprofloxacin-d8	CFX-d8	3.865	340.2	322.2	235	113
Roxithromyxcin-d7	RTM-d7	11.48	845.6	158.1	116	136
Caffeine-13C3	Caffeine-13C3	3.733	198.1	140	112.1	84

**Table A2:** Description of recovery, limits of quantification (LOQ) and detection (LOD).

Group	Compound	Recovery (%)		LOD (ng L^−1^)	LOQ (ng L^−1^)
		50 ng L^−1^	100 ng L^−1^		
Fluoroquinolones	CFX	65.0 ± 11.4	72.8 ± 13.0	0.040	0.122
	EFX	70.8 ± 10.5	58.9 ± 6.8	0.316	0.957
	OFX	70.7 ± 14.0	79.4 ± 7.2	0.023	0.069
	OA	96.0 ± 1.6	75.9 ± 11.6	0.008	0.024
	SAR	89.9 ± 7.0	92.7 ± 15.3	0.024	0.072
	SFX	78.7 ± 9.1	95.1 ± 15.4	0.028	0.084
	NFX	66.2 ± 13.7	65.9 ± 7.6	0.067	0.203
Tetracycline	CTC	118.2 ± 16.2	91.5 ± 14.5	0.210	0.640
	DC	91.03 ± 7.1	76.41 ± 15.9	0.226	0.685
	OTC	107.6 + 13.9	63.69 + 9.1	0.201	0.610
	TC	89.27 + 6.7	72.79 + 21.3	0.230	0.696
Macrolides	TYL	80.17 + 4.3	65.18 + 11.6	0.013	0.041
	RTM	68.0 + 5.4	60.33 + 6.1	0.041	0.126
	CTM	82.35 + 1.3	76.5 + 4.1	0.091	0.275
Sulfonamides	STZ	90.2 ± 19.3	76.8 ± 12.3	0.018	0.056
	SMM	98.4 ± 5.4	91.8 ± 10.6	0.108	0.328
	SAZ	70.0 ± 7.0	87.6 ± 3.5	0.110	0.334
	SMX	96.2 ± 2.0	90.0 ± 5.3	0.056	0.170
	SMZ	80.2 ± 6.7	80.7 ± 14.8	0.017	0.052
	SDZ	82.4 ± 6.4	99.3 ± 10.7	0.051	0.155
Diaminopyrimidines	TMP	95.1 ± 4.7	99.8 ± 7.8	0.011	0.033
